# The case of Montréal's missing food deserts: Evaluation of accessibility to food supermarkets

**DOI:** 10.1186/1476-072X-6-4

**Published:** 2007-02-12

**Authors:** Philippe Apparicio, Marie-Soleil Cloutier, Richard Shearmur

**Affiliations:** 1 Spatial Analysis and Regional Economics Laboratory, Institut national de la recherche scientifique – Urbanisation, Culture et Société, 385 rue Sherbrooke est, Montréal (Québec), H2X 1E3, Canada; 2 Department of Geography, University of Montréal, Pavillon Strathcona, 520 Côte Sainte-Catherine, Outremont (Québec), H2V 2B8, Canada

## Abstract

**Background:**

Access to varied, healthy and inexpensive foods is an important public health concern that has been widely documented. Consequently, there is an increasing interest in identifying food deserts, that is, socially deprived areas within cities that have poor access to food retailers. In this paper we propose a methodology based on three measures of accessibility to supermarkets calculated using geographic information systems (GIS), and on exploratory multivariate statistical analysis (hierarchical cluster analysis), which we use to identify food deserts in Montréal.

**Results:**

First, the use of three measures of accessibility to supermarkets is very helpful in identifying food deserts according to several dimensions: proximity (distance to the nearest supermarket), diversity (number of supermarkets within a distance of less than 1000 metres) and variety in terms of food and prices (average distance to the three closest different chain-name supermarkets).

Next, the cluster analysis applied to the three measures of accessibility to supermarkets and to a social deprivation index demonstrates that there are very few problematic food deserts in Montréal. In fact, census tracts classified as socially deprived and with low accessibility to supermarkets are, on average, 816 metres away from the nearest supermarket and within 1.34 kilometres of three different chain-name supermarkets.

**Conclusion:**

We conclude that food deserts do not represent a major problem in Montréal. Since geographic accessibility to healthy food is not a major issue in Montréal, prevention efforts should be directed toward the understanding of other mechanisms leading to an unhealthy diet, rather than attempting to promote an even spatial distribution of supermarkets.

## Background

Since the mid-1990s there has been an increasing interest–particularly in Britain–in identifying areas within cities that have poor access to basic retail services and, more specifically, to food retailers [1]. Such areas are known as food deserts, a concept defined by the UK Low Income Project Team as "areas of relative exclusion where people experience physical and economic barriers to accessing healthy food" [2](p.138). These barriers are often linked to spaces of poverty, in part because some people in these areas have little mobility, whether this be short-term (no access to a car) or long-term mobility (lack of choice of residence due to lack of means). The absence of supermarkets in some spaces of poverty would therefore suggest that low income families without a car will tend to shop in small local shops that often sell a smaller variety of foods, and at higher prices [3,4]. In other words, people's dietary choices may be influenced by the availability and type of food stores [5,6]. Implications of such associations for public policies lie in the relationship between poor access to healthy, varied and affordable food and poor health, even after controlling for individual socioeconomic factors [7,8]. This relationship, often seen in US cities, contributes to inequalities in health and may exacerbate equity and public health issues linked to socioeconomic deprivation [4,9,10]. Moreover, living under food insecurity has socio-familial (modified eating patterns, disrupted household dynamics) and psychological consequences (distress, guilt), no matter what the primary source of this insecurity may be: physical inaccessibility or monetary constraints, for example [11,12].

The interest in identifying and describing food deserts recently peaked with a special issue of *Urban Studies *devoted to "Food Deserts in British Cities" (October 2002). However, there is no clear agreement on what measures are relevant in identifying food deserts, which is contributing lately to debate about their actual existence, especially in the UK [13,14]. Notwithstanding this, there are few quantitative studies that focus on the identification and description of food deserts. Methodology for such research is often based on a single, relatively simple accessibility measure such as the number (or proportion/ratio per area or per population) of food retailers in a neighbourhood [3,5,15,16], the number of food retailers within a radius of *n *metres [17,18], or the minimum distance to the nearest food retailers [8]. Other research has improved various aspects of the way in which access to food is measured by developing more complex methodologies that include different and combined measures [19,20]. Recently as well, a series of exploratory analyses that refine measures of spatial accessibility to urban services and amenities un general have been published by urban planners and geographers [21-29].

In this paper we develop, apply and assess a more refined methodology for the identification of food deserts through geographic accessibility measures. We argue that since each accessibility measure corresponds to a different dimension of the food desert concept, no single measure can fully describe accessibility to food retailers. Consequently, three different accessibility measures are explored in the context of Montréal. The approach is based on spatial analytical techniques in a GIS environment and on multi-dimensional exploratory data analysis. The objective of this paper is to verify the presence of food deserts in Montréal, since, as mentioned by Smoyer-Tomic et al. [20], "Canada shares some similarities with the US and UK, yet its geographic, demographic, political and economic characteristics suggest that in terms of food access, its experience may be unique."

## Data

### Study area

This study focuses on the Island of Montréal which has 1.8 million inhabitants and is part of the Montréal census metropolitan area (CMA), which in turn has a population of about 3.4 millions. Social deprivation is an important concern in Montréal since, in 2000, 29% of its population lived under the low income thresholds defined by Statistics Canada. Consequently, accessibility to services and facilities, and in particular to healthy food, is an important social equity issue.

### Supermarket data

Supermarkets were defined as grocery stores associated with one of the seven major chains in Quebec: IGA, Intermarché, Loblaws, Maxi, Métro, Provigo and Super C. Information on their locations was gathered in June 2004 from a yellow pages directory and from the different chains' web sites. Addresses and affiliation were confirmed by telephoning the stores. In total, 167 supermarkets were integrated within a geographic information system (ArcGis) by geocoding addresses. Only the abovementioned affiliated supermarkets were retained for two reasons: 1) they represent a type of food retailer where the variety of products is greater and the pricing more competitive than in small grocery shops [8]; and 2) in Montréal, supermarkets represent approximately 24% of food retail outlets but 80% of food sales [3]. Morland et al. [5] also reported in 2002 that supermarkets had between two and four times the average number of "heart-healthy" foods compared with neighbourhood grocery and convenience stores. The choice to take into account the geographic position of supermarkets but not their characteristics could be considered as a limitation in our study. In fact, supermarkets can vary greatly in terms of floor areas and quality of products but these variables are not taken into account in our accessibility measures, and neither are potential qualitative data related to purchasing behaviour.

### Low income population data and social deprivation index

In order to relate supermarket accessibility and deprivation, two variables were used at the census tract level: low income population and a social deprivation index. The first variable identifies the population belonging to low income households. These people allot 20% more than the rest of Canadian households to three basic needs: housing, food and clothing [30]. This variable refers to the *relative deprivation *concept introduced by Townsend [31], where deprivation is present when living conditions are below those of the majority in a given population.

However, to evaluate urban deprivation at the census tract level, the use of a single variable such as low income population is not enough. Social deprivation is also associated with other individual characteristics identified as factors contributing to deprivation or dimensions of deprivation, such as, for example: belonging to a lone-parent family, unemployment, low level of schooling or recent immigration [30,32,33]. Consequently, a status of deprivation cannot be related to one characteristic alone, but more often to an accumulation of many. Therefore, to characterize the distribution of social deprivation across the Island of Montréal, a social deprivation index was calculated based on five types of populations that are usually associated with poverty.

The index represents the sum of five variables collected at the census tract level and standardized on a 0 to 1 scale: i) the percentage of people with low incomes relative to the total population; ii) the percentage of lone-parent families relative to the total number of families; iii) the unemployment rate; iv) the percentage of individuals over the age of 20 with no more than Grade 9 education; and v) the percentage of recent immigrants (immigrants who had arrived between 1996 and 2001) relative to the total population (Table 1). The deprivation index can vary between 0 (minimum deprivation) and 5 (maximum deprivation). As we might expect, these five variables are positively intercorrelated, but the fact that they are all included in one index allows us to locate census tracts where there is an accumulation of more than one variable relating to deprivation (see Table 2). It is also important to mention that the percentage of recent immigrants was included in the index because recent studies have shown a significant relation between urban poverty and immigration in Canada [30,34-36].

**Table 1:** Descriptive statistics of social deprivation variables in census tracts on the Island of Montréal

	**Low income population (%)**	**Lone-parent families (%)**	**Unemployment rate**	**Adults with low level of schooling (%)**	**Recent Immigrants (%)**	**Social deprivation index**
Mean	29.98	21.38	9.46	14.63	5.20	1.56
Std deviation	14.27	7.84	4.55	8.46	4.68	0.61
CV*	0.48	0.37	0.48	0.58	0.90	0.39
Skewness	0.36	0.32	2.07	0.26	1.89	0.29
Kurtosis	-0.12	0.54	10.96	-0.75	3.91	0.09
Minimum	1.23	0.00	0.00	0.00	0.00	0.17
Maximum	82.64	51.28	47.44	37.05	25.79	3.90
Percentiles						
5%	8.08	8.73	3.96	1.90	0.53	0.59
10%	11.44	11.33	4.92	3.31	1.02	0.75
25% Q1	19.68	16.01	6.59	7.54	2.11	1.16
50% Median	28.70	21.23	8.56	14.23	3.85	1.55
75% Q3	39.81	26.25	11.67	20.99	6.49	1.94
90%	49.90	31.27	14.99	26.28	10.75	2.32
95%	53.78	35.19	16.99	28.71	16.11	2.68

* Coefficient of variation.

**Table 2:** Pearson correlations for social deprivation variables in census tracts on the Island of Montréal

	(a)	(b)	(c)	(d)	(e)
(a) Low income population (%)	1.000				
(b) Lone-parent families (%)	0.677*	1.000			
(c) Unemployment rate	0.760*	0.498*	1.000		
(d) Adults with low level of schooling (%)	0.492*	0.522*	0.420*	1.000	
(e) Recent immigrants (%)	0.489*	0.083	0.502*	-0.025	1.000

* significant at the 0.01 level (2-tailed).

In this study, the use of the two variables – low income and deprivation index – meets two different objectives. Firstly, the low income population variable allows us to verify whether or not poor people have good accessibility to supermarkets compared with the rest of the population. Secondly, the social deprivation index enables us to relate deprivation and accessibility for each census tract and to thus identify food deserts, that is, urban spaces where deprivation is high and accessibility is low.

## Methods and data analysis

### Measuring accessibility to supermarkets

Although not often applied in the context of food deserts, the evaluation of accessibility to urban amenities (schools, green spaces, etc.) has been conducted using methodologies relying on rigorously defined accessibility measures calculated within geographic information systems (GIS) [23,24,26,37-39].

The most commonly used measures of accessibility found in the literature are the gravity model, the mean distance to all services, the distance to the closest service and the mean distance to all the services included within an *n *metre radius [22,24,26,40,41]. Talen and Anselin [24] and Talen [41] have shown that the choice of the accessibility measure is fundamental since, with a given set of data, accessibility varies depending on the indicator used. Consequently, we believe that using only one measure provides a poor description of a given population's accessibility to a particular service: on the other hand, using several different measures allows one to adequately describe the complexity of a population's accessibility to a service.

Three different measures of accessibility are retained here: distance to the closest supermarket (Equation 1), in order to evaluate immediate proximity; number of supermarkets within a walkable distance of less than 1000 metres (approximately a 15-minute walk for an adult in an urban setting) [42] (Equation 2), in order to evaluate the diversity provided by the immediate surroundings; and mean distance to three supermarkets belonging to different companies (Equation 3), in order to evaluate access to variety in terms of both products and prices. This last measure is based on the hypothesis that different supermarket companies have numerous brands for the same product and a range of retail and discount prices, thus increasing the variety of choice for customers.

To better account for the spatial distribution of population (that is, to minimize aggregation error [25]), and notwithstanding the fact that our analysis is at the census tract (CT) level, the three accessibility measures were calculated from the centroid of blocks. Then, to obtain accessibility data at the CT level, we calculate the population-weighted average measure of blocks within each CT's boundaries:

Zia∑b∈iwb(min⁡|dbs|)∑b∈iwb,     (Equation 1)
 MathType@MTEF@5@5@+=feaafiart1ev1aaatCvAUfKttLearuWrP9MDH5MBPbIqV92AaeXatLxBI9gBaebbnrfifHhDYfgasaacH8akY=wiFfYdH8Gipec8Eeeu0xXdbba9frFj0=OqFfea0dXdd9vqai=hGuQ8kuc9pgc9s8qqaq=dirpe0xb9q8qiLsFr0=vr0=vr0dc8meaabaqaciaacaGaaeqabaqabeGadaaakeaacqWGAbGwdaqhaaWcbaGaemyAaKgabaGaemyyaegaaOWaaSaaaeaadaaeqbqaaiabdEha3naaBaaaleaacqWGIbGyaeqaaOGaeiikaGIagiyBa0MaeiyAaKMaeiOBa42aaqWaaeaacqWGKbazdaWgaaWcbaGaemOyaiMaem4CamhabeaaaOGaay5bSlaawIa7aiabcMcaPaWcbaGaemOyaiMaeyicI4SaemyAaKgabeqdcqGHris5aaGcbaWaaabuaeaacqWG3bWDdaWgaaWcbaGaemOyaigabeaaaeaacqWGIbGycqGHiiIZcqWGPbqAaeqaniabggHiLdaaaOGaeiilaWIaaCzcaiaaxMaadaqadaqaaiabbweafjabbghaXjabbwha1jabbggaHjabbsha0jabbMgaPjabb+gaVjabb6gaUjabbccaGiabigdaXaGaayjkaiaawMcaaaaa@60C8@

Where:

Zia
 MathType@MTEF@5@5@+=feaafiart1ev1aaatCvAUfKttLearuWrP9MDH5MBPbIqV92AaeXatLxBI9gBaebbnrfifHhDYfgasaacH8akY=wiFfYdH8Gipec8Eeeu0xXdbba9frFj0=OqFfea0dXdd9vqai=hGuQ8kuc9pgc9s8qqaq=dirpe0xb9q8qiLsFr0=vr0=vr0dc8meaabaqaciaacaGaaeqabaqabeGadaaakeaacqWGAbGwdaqhaaWcbaGaemyAaKgabaGaemyyaegaaaaa@30BC@ = mean distance between census tract and nearest supermarket.

*d*_*bs *_= distance between block centroid and supermarket *s*.

*w*_*b *_= total population of block *b *(entirely included in census tract *i*).

Zib=∑b∈iwb∑j∈SSj∑b∈iwb,     (Equation 2)
 MathType@MTEF@5@5@+=feaafiart1ev1aaatCvAUfKttLearuWrP9MDH5MBPbIqV92AaeXatLxBI9gBaebbnrfifHhDYfgasaacH8akY=wiFfYdH8Gipec8Eeeu0xXdbba9frFj0=OqFfea0dXdd9vqai=hGuQ8kuc9pgc9s8qqaq=dirpe0xb9q8qiLsFr0=vr0=vr0dc8meaabaqaciaacaGaaeqabaqabeGadaaakeaacqWGAbGwdaqhaaWcbaGaemyAaKgabaGaemOyaigaaOGaeyypa0ZaaSaaaeaadaaeqbqaaiabdEha3naaBaaaleaacqWGIbGyaeqaaaqaaiabdkgaIjabgIGiolabdMgaPbqab0GaeyyeIuoakmaaqafabaGaem4uam1aaSbaaSqaaiabdQgaQbqabaaabaGaemOAaOMaeyicI4Saem4uamfabeqdcqGHris5aaGcbaWaaabuaeaacqWG3bWDdaWgaaWcbaGaemOyaigabeaaaeaacqWGIbGycqGHiiIZcqWGPbqAaeqaniabggHiLdaaaOGaeiilaWIaaCzcaiaaxMaadaqadaqaaiabbweafjabbghaXjabbwha1jabbggaHjabbsha0jabbMgaPjabb+gaVjabb6gaUjabbccaGiabikdaYaGaayjkaiaawMcaaaaa@5D6E@

Where:

Zib
 MathType@MTEF@5@5@+=feaafiart1ev1aaatCvAUfKttLearuWrP9MDH5MBPbIqV92AaeXatLxBI9gBaebbnrfifHhDYfgasaacH8akY=wiFfYdH8Gipec8Eeeu0xXdbba9frFj0=OqFfea0dXdd9vqai=hGuQ8kuc9pgc9s8qqaq=dirpe0xb9q8qiLsFr0=vr0=vr0dc8meaabaqaciaacaGaaeqabaqabeGadaaakeaacqWGAbGwdaqhaaWcbaGaemyAaKgabaGaemOyaigaaaaa@30BE@ = mean number of supermarkets within 1000 m of census tract population.

*S *= all supermarkets.

*S*_*j *_= number of supermarkets within 1000 m of the block centroid (*d*_*bs *_< 1000).

*w*_*b *_= total population of block *b *(entirely included in census tract *i*).

Zic=∑b∈iwb∑sdbsn∑b∈iwb,     (Equation 3)
 MathType@MTEF@5@5@+=feaafiart1ev1aaatCvAUfKttLearuWrP9MDH5MBPbIqV92AaeXatLxBI9gBaebbnrfifHhDYfgasaacH8akY=wiFfYdH8Gipec8Eeeu0xXdbba9frFj0=OqFfea0dXdd9vqai=hGuQ8kuc9pgc9s8qqaq=dirpe0xb9q8qiLsFr0=vr0=vr0dc8meaabaqaciaacaGaaeqabaqabeGadaaakeaacqWGAbGwdaqhaaWcbaGaemyAaKgabaGaem4yamgaaOGaeyypa0ZaaSaaaeaadaaeqbqaaiabdEha3naaBaaaleaacqWGIbGyaeqaaaqaaiabdkgaIjabgIGiolabdMgaPbqab0GaeyyeIuoakmaaqafabaWaaSaaaeaacqWGKbazdaWgaaWcbaGaemOyaiMaem4CamhabeaaaOqaaiabd6gaUbaaaSqaaiabdohaZbqab0GaeyyeIuoaaOqaamaaqafabaGaem4DaC3aaSbaaSqaaiabdkgaIbqabaaabaGaemOyaiMaeyicI4SaemyAaKgabeqdcqGHris5aaaakiabcYcaSiaaxMaacaWLjaWaaeWaaeaacqqGfbqrcqqGXbqCcqqG1bqDcqqGHbqycqqG0baDcqqGPbqAcqqGVbWBcqqGUbGBcqqGGaaicqaIZaWmaiaawIcacaGLPaaaaaa@5DDC@

Where:

Zic
 MathType@MTEF@5@5@+=feaafiart1ev1aaatCvAUfKttLearuWrP9MDH5MBPbIqV92AaeXatLxBI9gBaebbnrfifHhDYfgasaacH8akY=wiFfYdH8Gipec8Eeeu0xXdbba9frFj0=OqFfea0dXdd9vqai=hGuQ8kuc9pgc9s8qqaq=dirpe0xb9q8qiLsFr0=vr0=vr0dc8meaabaqaciaacaGaaeqabaqabeGadaaakeaacqWGAbGwdaqhaaWcbaGaemyAaKgabaGaem4yamgaaaaa@30C0@ = mean distance between census tract population and *n *different chain-name supermarkets.

*d*_*bs *_= distance between block centroid and supermarket *s*; *d*_*bs *_is sorted in ascending order.

*n *= number of different chain-name supermarkets to be included in measure (here *n *= 3).

*w*_*b *_= total population of block *b *(entirely included in census tract *i*).

The three accessibility measures are calculated using the shortest network distance, which closely corresponds to the shortest path for going to a supermarket on foot. Network distances are based on CanMap Streetfiles from DMTI [43] and are computed with the Network Analyst extension of ArcView 3.3 [44].

### Linking low income population, level of social deprivation and accessibility to supermarkets

After having identified socially deprived areas and areas with high and low levels of accessibility to food retailers in Montréal, we used an empirical approach to study the link between accessibility and a neighbourhood's socioeconomic status. Three different approaches are used to explore this link across the 506 census tracts: (1) calculation of population-weighted descriptive accessibility statistics, in order to compare low income people's accessibility to supermarkets relative to the rest of the population, (2) calculation of Pearson correlation coefficients to explore the statistical significance of the link between social deprivation and supermarket accessibility, and finally, (3) computation of a hierarchical cluster analysis [45] to classify and characterize census tracts in different groups of CTs with similar levels of social deprivation and accessibility. In this last exploratory step, it should be possible to identify potential food deserts, that is, CTs which combine social deprivation with low accessibility to supermarkets, but it will also be possible to point out deprived areas with good accessibility. The objective of the hierarchical cluster analysis is not only to locate food deserts but also to categorize all CTs in terms of deprivation and accessibility.

## Results

### Mapping low income population and social deprivation

**Figure 1:**
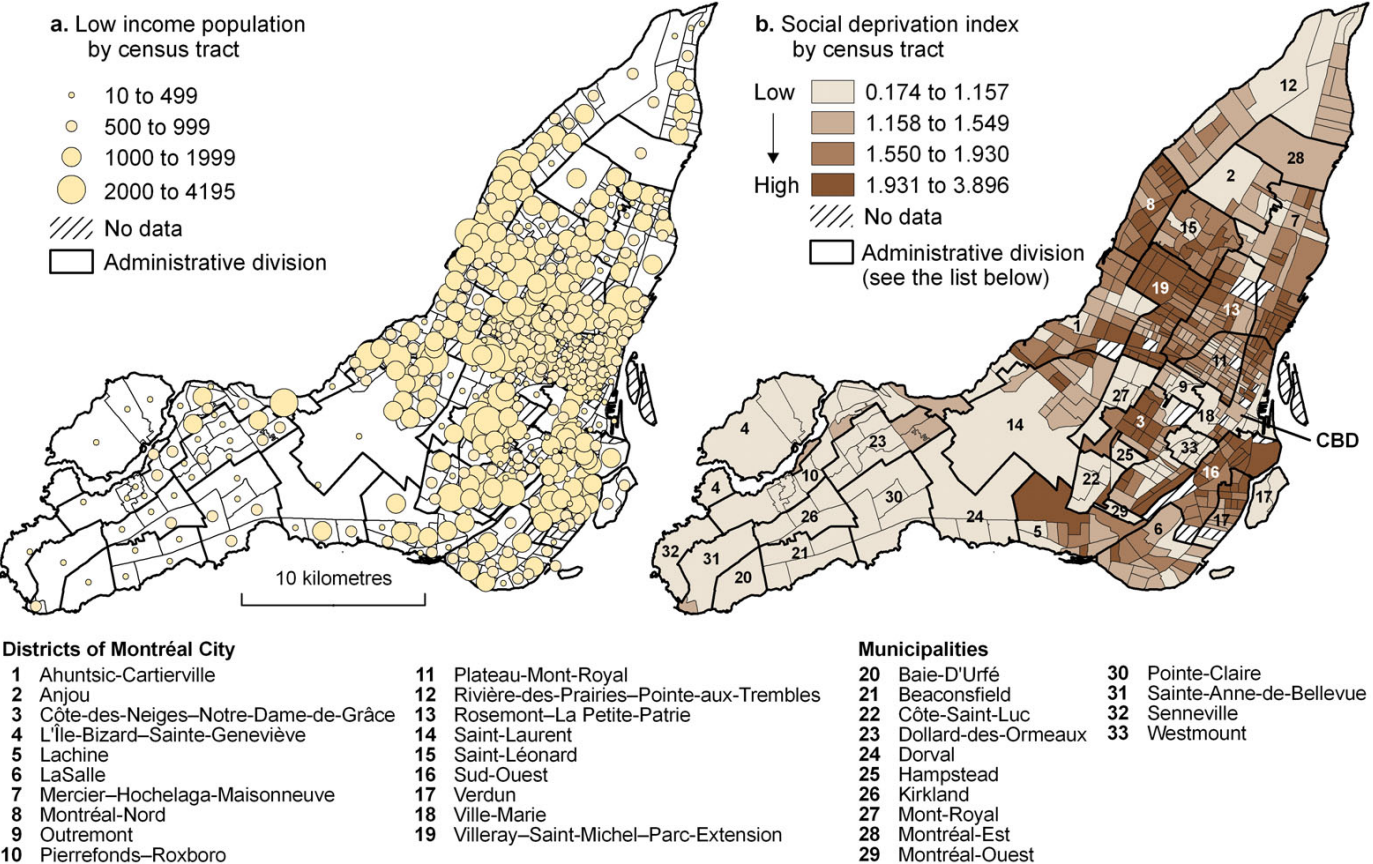
Spatial distribution of low income population and social deprivation index on the Island of Montréal, 2001.

In Montréal, low income people live mostly in the centre of the island and in the close periphery surrounding the CBD, while they are almost absent in far eastern and western boroughs (Figure 1a). Mapping the social deprivation index for the year 2001 confirms past analyses of the distribution of poverty within the agglomeration (CMA) of Montréal at the census tract level (Figure 1b). Indeed, whatever the method used, whether mapping low income populations [46,47], using a deprivation index [48,49], or performing factor analysis on various socioeconomic indicators [50,51], the results are similar: the most socially deprived census tracts are located within the City of Montréal (see Figure 1 for municipality locations), and specifically in the south of Mercier-Hochelaga-Maisonneuve (district 7), across most of Sud-Ouest (district 16), to the north and southeast of Côte-des-Neiges-Notre-Dame-de-Grâce (district 3), across most of Villeray-Saint-Michel-Parc-Extension (district 19) and to the north and east of Ville-Marie (district 18).

### Mapping the three measures of accessibility

**Figure 2:**
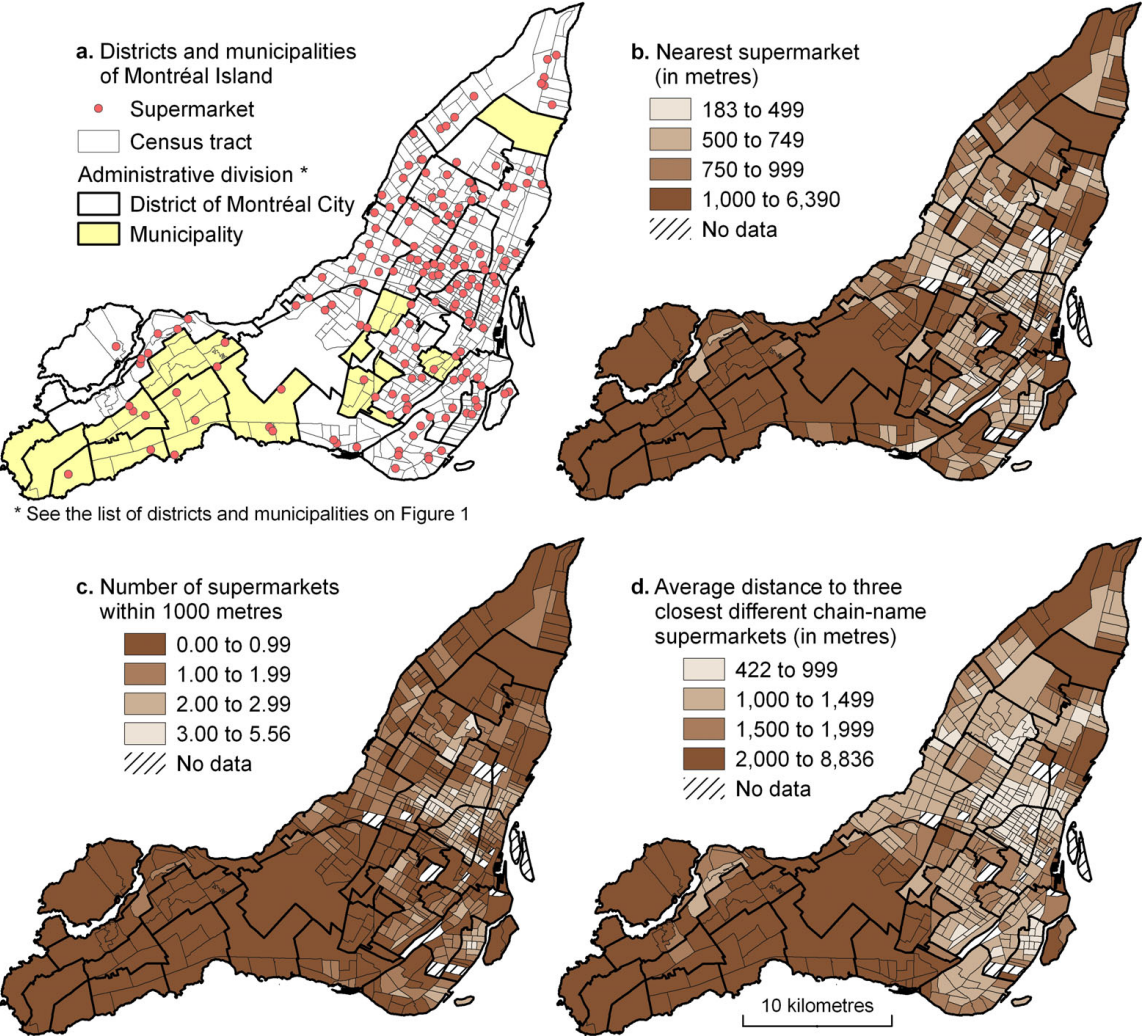
Spatial distribution of supermarket accessibility measures on the Island of Montréal, 2001.

Mapping the three accessibility measures results in two main observations (Figures 2b, 2c and 2d). Overall, accessibility to supermarkets decreases as one moves out from central neighbourhoods to peripheral areas. This observation is particularly striking for two of the measures: number of supermarkets within a 1000 metre radius (diversity measure) and mean distance to the three closest different chain-name supermarkets (variety measure) (Figures 2c and 2d). In fact, spatial autocorrelation statistics are higher for these two measures: Moran's I [52,53] values are higher at 0.72 and 0.63 as opposed to 0.54 for the nearest supermarket indicator (Table 3). This implies that, for this last measure where Moran's I is lower, areas with similar values are less clustered in space.

**Table 3:** Spatial autocorrelation statistics for social accessibility measures

	Moran's I *	**z-score**
Nearest supermarket (in metres)	0.54	21.68
Number of supermarkets within 1000 metres	0.72	28.36
Average distance to three closest different chain-name supermarkets (in metres)	0.63	25.37

* calculated with a queen binary connectivity matrix (1 where census tracts *i *and *j *are contiguous; 0 otherwise).

Indeed, there are isolated census tracts in peripheral districts of the City of Montréal that have good accessibility to one supermarket (less than 500 metres, Figure 2a). This leads to a second observation: in peripheral areas, good accessibility tends to be synonymous with proximity to a single supermarket (good proximity), whereas in central areas, good accessibility is associated with the presence of several different supermarkets in the immediate surroundings (good diversity and variety).

### Relating low income population, social deprivation and accessibility measures

#### Level of accessibility for the low income population: some weighted descriptive statistics

Before mapping out food deserts at the intra-urban level, one needs to verify whether or not the low income population has good access to supermarkets, compared with the rest of the population. To do this, we computed population-weighted descriptive statistics for each accessibility measure (Table 4). These statistics demonstrate that the low income population in fact has *better *accessibility than the rest of the population. The statistics also show that for low income people, accessibility to supermarkets does not seem to be a problem: 50% of low income individuals live less than 683 metres from a supermarket and 75% of them live less than 947 metres away (see median and Q3 in Table 4).

**Table 4:** Descriptive statistics for accessibility measures weighted by populations

	**Nearest supermarket (in metres)**	**Number of supermarkets within 1000 metres**	**Average distance to three closest different chain-name supermarkets (in metres)**
**Weight: Low income population**			
Mean	792.99	1.28	1360.84
Std deviation	14279.38	28.91	19794.74
Coef. of variation	18.01	22.51	14.55
Percentiles			
5%	335.59	0.00	716.66
10%	378.37	0.20	804.47
25% Q1	519.93	0.62	1010.81
50% Median	682.90	1.07	1227.00
75% Q3	946.93	1.86	1509.37
90%	1254.99	2.51	2114.87
95%	1619.21	2.92	2540.54

**Weight: No low income population**			
Mean	994.92	1.04	1617.33
Std deviation	33098.02	44.73	42143.97
Coef. of variation	33.27	43.18	26.06
Percentiles			
5%	351.58	0.00	763.68
10%	439.02	0.00	883.39
25% Q1	574.08	0.34	1097.31
50% Median	838.89	0.90	1386.49
75% Q3	1135.15	1.46	1883.18
90%	1751.91	2.25	2727.93
95%	2284.80	2.84	2996.09

#### Relation between social deprivation and accessibility measures: correlation analysis

Even though descriptive statistics suggest that the low income population has better access to supermarkets, is there a statistically significant link between social deprivation and people's accessibility to supermarkets in Montréal? Again, values of the Pearson coefficients of correlation between accessibility and social deprivation show that socially deprived CTs have significantly better access to supermarkets (Table 5), but these values are relatively weak (between -0.5 and 0.5). These results reflect the fact that all of these variables display high levels of variability across CTs; they can also mean that socially deprived areas with good (bad) accessibility coexist with non-deprived areas with good (bad) accessibility. It is the deprived *and *inaccessible areas that need to be identified, since it is people in these areas who may be negatively affected by the lack of accessibility to supermarkets. In order to explore this, hierarchical cluster analysis [45] was performed on the 506 CTs, in classifying each CT according to its three accessibility measures and its deprivation index. In this way, we could identify groups of CTs with similar profiles of accessibility and deprivation, which could then be analyzed in order to ascertain whether there were any combinations that could be interpreted as food deserts. From a methodological perspective it could be argued that undue weight is given to accessibility in this cluster analysis, since it covers three accessibility measures but only one social deprivation measure. Given that the focus of the study is to explore the different dimensions of accessibility, and that the results of the weighted cluster analysis (reducing the weight of each accessibility measure to 1/3) are similar to those presented, we have chosen to retain the unweighted results.

**Table 5:** Pearson correlations between accessibility measures and social deprivation index

**Accessibility measure**	***Social deprivation index***
Nearest supermarket	-0.426
Number of supermarkets within 1000 metres	0.285
Average distance to three closest different chain-name supermarkets	-0.425

Note: all values are significant at p < 0.001 level

#### A typology of census tracts according to levels of social deprivation and measures of accessibility to supermarkets: cluster analysis

**Figure 3:**
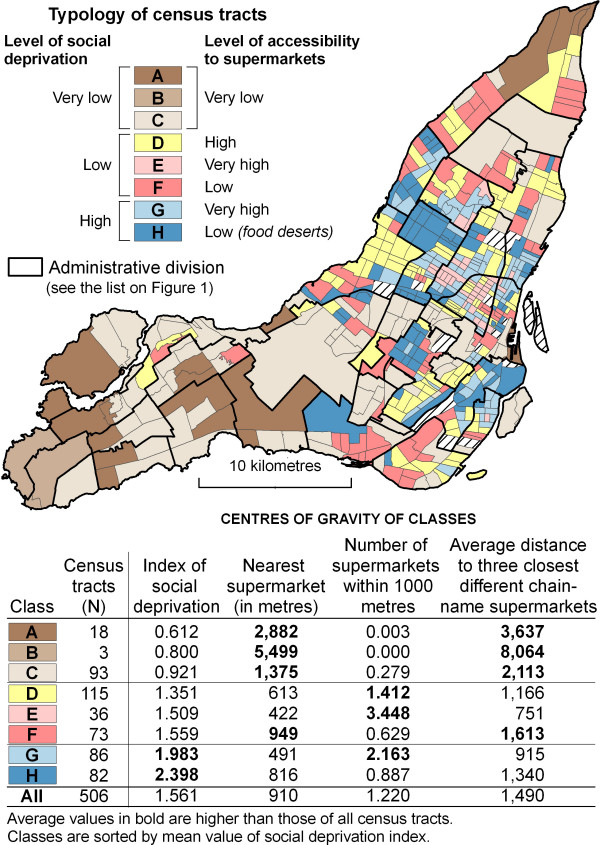
Typology of census tracts on the basis of a social deprivation index and measures of accessibility to supermarkets on the Island of Montréal, 2001.

Eight types of CTs are identified from the cluster analysis (Figure 3). Most of the CTs classified in A, B and C are located in the western and eastern parts of the Island of Montréal; they represent typical suburban areas with very low levels of social deprivation and also very low levels of accessibility to supermarkets. For example, the population living in the type-B CTs is on average 5.5 kilometres away from the nearest supermarket, and the average distance to the three closest different chain-name supermarkets is 8 kilometres. However, this very low accessibility is not problematic, since it can be assumed that most of the resident population has chosen to live there and has access to a car for food shopping.

**Figure 4:**
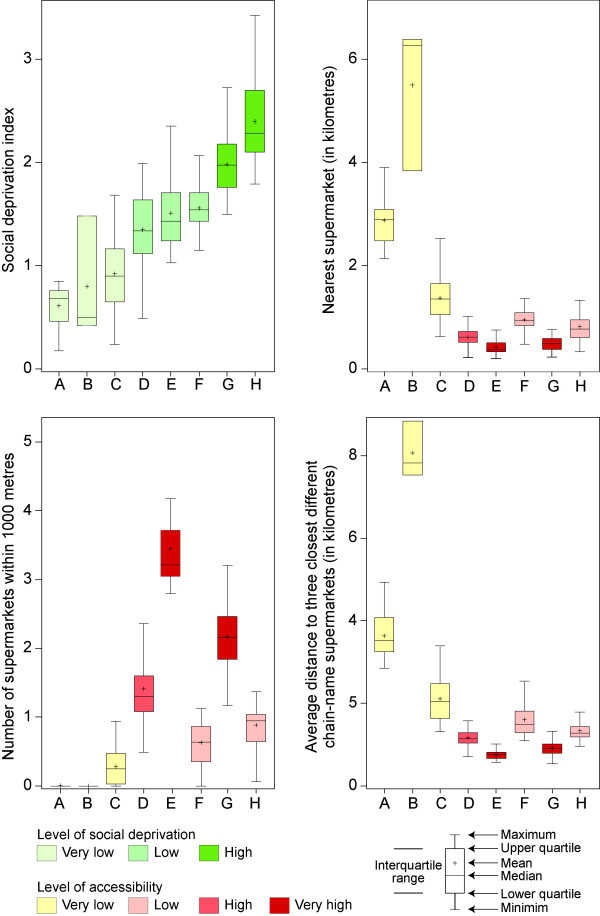
Boxplot of social deprivation and accessibility measures for classes of census tracts (see Figure 3).

CTs of the next three types (D, E, F) are characterized by low levels of social deprivation, but also by different levels of accessibility (Figures 3 and 4). The highest level of accessibility is observed in type E, which includes 36 CTs located in central, gentrified neighbourhoods such as Plateau-Mont-Royal. On average, the population living in these CTs is 422 metres away from the nearest supermarket (very good proximity), has 3.44 supermarkets within a 1000 metre radius (very good diversity), and is on average 751 metres away from three different chain-name supermarkets (very good variety).

The last two types, or classes, are rather socially deprived CTs (classes G and H, Figures 3 and 4). As in the previously described class E, CTs in class G have high levels of accessibility over all three dimensions–proximity, diversity and variety (Figure 4). On the other hand, the 82 class-H CTs are potentially problematic, with a high level of social deprivation and a low level of accessibility to supermarkets. These CTs, which are potential food deserts, are located in Montréal-Nord (district 8), in Saint-Michel and Parc-Extension (district 19), in the Sud-Ouest (district 16) and in the boroughs of Côte-des-Neiges (district 3), Hochelaga-Maisonneuve and Ville-Marie (districts 7 and 18) (Figure 3). The problematic nature of these CTs ought, however, to be relativized. On average, the population located in these CTs is 816 metres away from the nearest supermarket, that is, about a 10-minute walk, and the average distance to the three closest different chain-name supermarkets is 1340 metres. Although it can be difficult for older people or people with a lot of shopping bags to travel these distances, they are still reasonable from a typical adult point of view. In fact, they do not differ greatly from the distances observed in the clusters considered to have the best accessibility to supermarkets (for example, type E: 422 metres to the nearest supermarket and 751 metres to the three closest chain supermarkets; and type G: 491 metres and 915 metres respectively). In fact, the main characteristic of type-H census tracts is that they have fewer supermarkets in their immediate vicinity (on average 0.89 within a 1000 metre radius).

If we look at the proportion of the total population residing in the eight types of CTs identified (Table 6), we can see that 58.41% of Island of Montréal inhabitants live in spaces with very low or low accessibility to supermarkets (classes A, B, C, F and H), and that 17.18% of these also live in potential food deserts, that is, spaces that are both socially deprived *and *with low accessibility to supermarkets (class H). A more reassuring observation is that 15.57% of the population living in spaces of deprivation nevertheless benefits from good accessibility to supermarkets (class G).

**Table 6:** Total and low income populations living in the eight types of CTs identified according to social deprivation and accessibility to supermarkets

			**Total population**		**Low income population**		
**Class**	**Level of social deprivation**	**Level of accessibility to supermarkets**	N	%	N	% ▼	% ▶
**A**	Very low	Very low	65312	3.61	5405	1.05	8.46
**B**	Very low	Very low	6032	0.33	845	0.16	15.53
**C**	Very low	Very low	390386	21.58	58705	11.43	15.32

**D**	Low	High	386785	21.38	94090	18.31	24.83
**E**	Low	Very High	84029	4.65	26290	5.12	32.11
**F**	Low	Low	283997	15.70	76270	14.85	27.62

**G**	High	Very High	281576	15.57	110925	21.59	40.03
**H (food deserts)**	High	Low	310754	17.18	141235	27.49	46.46

Total			1808871	100.00	513765	100.00	29.01

## Discussion

From a methodological viewpoint, three different accessibility measures using the shortest network distance were calculated: nearest supermarket (proximity), number of supermarkets within 1000 metres (diversity), and average distance to the three closest different chain-name supermarkets (variety). Our analysis of these three indicators has shown that the use of several accessibility measures provides different results for each census tract. For instance, a tract may show accessibility in terms of proximity, but may show inaccessibility in terms of variety and diversity. Consequently, our results emphasize the fact that it is important to identify food deserts using more than one indicator: furthermore, it is useful to analyze these indicators separately, since each one measures a different dimension of food deserts.

The empirical results for Montréal demonstrate the usefulness of this approach, and prompt two observations. Firstly, there are very few problematic food deserts on the Island of Montréal: this is in keeping with the results of Cummins and Macintyre for Glasgow [15] and Smoyer-Tomic et al. for Edmonton [20]. In Montréal, those tracts classified as deprived *and *with low accessibility to supermarkets are, on average, 816 metres away from the nearest supermarket, and within 1.34 kilometres of three different chain-name supermarkets. These "potential food deserts" are mostly isolated cases and do not represent a city-wide public health issue. Although six inhabitants out of ten have low accessibility to supermarkets (58.41%), less than two out of ten live in areas that are defined both as poor and as food deserts (17.18%).

The second and related observation is that accessibility to supermarkets decreases as one moves from central areas to peripheral neighbourhoods. This mirrors the tendency for incomes to increase along the same dimension [54], and is not surprising: it reflects the tendency for suburban development to be less dense, and preferred by middle income families, which generally have no problem accessing motorized transport. This suggests that supermarket providers in Montréal are responsive to the mobility of the local population and to population densities: low density and highly motorized areas tend to have fewer (but one presumes larger) supermarkets, whereas the more central – denser and less motorized – areas have more (but one presumes somewhat smaller) supermarkets. Although this spatial distribution of supermarkets does not pose problems at this time, issues may arise in suburban areas with low accessibility to supermarkets as the suburban population ages and loses mobility. Finally, it is worth noting that good accessibility in some suburban areas tends to involve proximity to a single supermarket, while in central areas it involves the presence of several different chain-name supermarkets in the immediate vicinity.

## Conclusion

It has been argued that identifying deprived areas with poor accessibility to food retailers is an important public health concern. Yet, we have shown that the identification of food deserts across a large city or metropolitan area is not straightforward for a variety of reasons. The accessibility measures used in this study cover three different dimensions of accessibility to food, but they are all based on geographic distance. However, recent studies increasingly demonstrate that access to food can be limited by several constraints, some of which are far more complex than geographic accessibility [9,13,14]. Social and cultural norms, physical disability, economic assets or attitude toward and knowledge about food and cooking are a few examples of non-geographic barriers to accessing good food. The existence of such barriers makes it difficult to evaluate the consequences of a geographic food desert in a community.

Even from a purely geographic perspective, supermarkets are not the only food retailers where good and healthy food can be bought. Without being unduly optimistic, other food retailers such as fruit and vegetable shops, specialty stores (butcher, fishmonger) and ethnic grocery shops may be present in deprived areas with poor accessibility to supermarkets. It is possible that purchases at these various small stores offer a range of healthy food products. The presence of smaller or independent grocery shops could thus fill the gap caused by the absence of supermarkets. Moreover, there can be differences in food availability, quality and in the price of products in these different types of food retailers aside from the supermarkets themselves. For example, Cummins and Macintyre [6] found that prices are up to four times lower in the cheapest stores compared with the most expensive retailers for a "range of foodstuffs comprising a modest but adequate diet." Since Montréal has not experienced the supermarket "redlining" of poorer districts seen elsewhere in the world, observations suggest that the numerous ethnic food shops may make a difference in multiethnic neighbourhoods such as Parc-Extension and Côte-des-Neiges. In other areas, such as Hochelaga-Maisonneuve and Saint-Henri, the paucity of alternative grocery stores apparently reinforces the existence of potential food deserts.

Conversely, geographic access to good food, while no doubt an enabling factor for a good diet, is by no means a guarantee. In fact, studies seem to be contradictory on this point: some find an association between supermarket proximity and better-quality diet and purchases [8,9], while others find no such relationship [55-57]. Now that there is a wide range of descriptive studies in the international literature on the presence (or absence) of food deserts, there is a need to improve our understanding of such a relationship between local environments, purchasing and diet behaviours and the local population's health status. This link, while often mentioned in the literature, is not well documented. Future studies in this regard will reinforce the potential to bring new and useful knowledge to this field.

Finally, since geographic accessibility to healthy food is not a major issue for most people in Montréal, research and prevention efforts in this urban area should be directed toward understanding other factors that can lead to an unhealthy diet. As Wrigley [4] pointed out, there is a need to understand, at the household and individual level, the experience of food retail access, regardless of whether it is poor or not, through detailed fieldwork including, for example, the obtaining of data on self-reported health status, mobility, accessibility or even coping mechanisms. Such research could also investigate the potential effects of a change in the foodscape on the residents' purchasing and diet behaviours as well as on their health. Policy interventions based on these results would then be able to more effectively improve the diet and health of urban residents.

## Competing interests

The author(s) declare that they have no competing interests.

## Authors' contributions

PA is the principal investigator of the study. He carried out the GIS, statistical and mapping analyses. MSC reviewed the literature and wrote parts of the paper. All authors jointly drafted and critically revised the paper, and read and approved the final manuscript.
